# Case report: Dupilumab leads to an increased chance of head and neck *Staphylococcus aureus* infection in atopic dermatitis patients

**DOI:** 10.3389/fmed.2023.1027589

**Published:** 2023-03-08

**Authors:** Min Chen, Kai Gao, Kamran Ali, Jinpeng Shan, YunMi Qiu, Tianci Xie, Yiling Yu, Liming Wu

**Affiliations:** ^1^Department of Dermatology, The Fourth School of Medicine Affiliated to Zhejiang Chinese Medical University, Hangzhou, Zhejiang, China; ^2^Department of Dermatology, Affiliated Hangzhou First People's Hospital, Zhejiang University School of Medicine, Hangzhou, China

**Keywords:** *Staphylococcus aureus* infection, adverse event, atopic dermatitis, dupilumab adverse reactions, drug reaction

## Abstract

Dupilumab was the first biological medication licensed to treat atopic dermatitis (AD), and it has shown remarkable effectiveness and safety in the treatment of moderate-to-severe atopic dermatitis. There are limited drug-related adverse events associated with dupilumab in atopic dermatitis (AD) treatment. Here, we present two cases of local *Staphylococcus aureus* infection during the treatment of atopic dermatitis with dupilumab.

## Introduction

Dupilumab was the first biological drug approved for atopic dermatitis (AD) treatment and has demonstrated significant effectiveness and safety in children and adults (1). Injection site reactions, eosinophilia, conjunctivitis, psoriasiform erythema, and herpes infection are the most commonly reported adverse events associated with dupilumab treatment for atopic dermatitis (2, 3). Dupilumab suppresses type 2 inflammation by inhibiting cytokine signaling, including IL-4 and IL-13. A clinical investigation demonstrated that interfering with IL-4R with dupilumab boosted microbial diversity and decreased Staphylococcus colonization in atopic dermatitis (4). Here, we present a case series of local *Staphylococcus aureus* infections during treatment of atopic dermatitis with dupilumab.

## Case presentation

### Case 1

**Figure 1:**
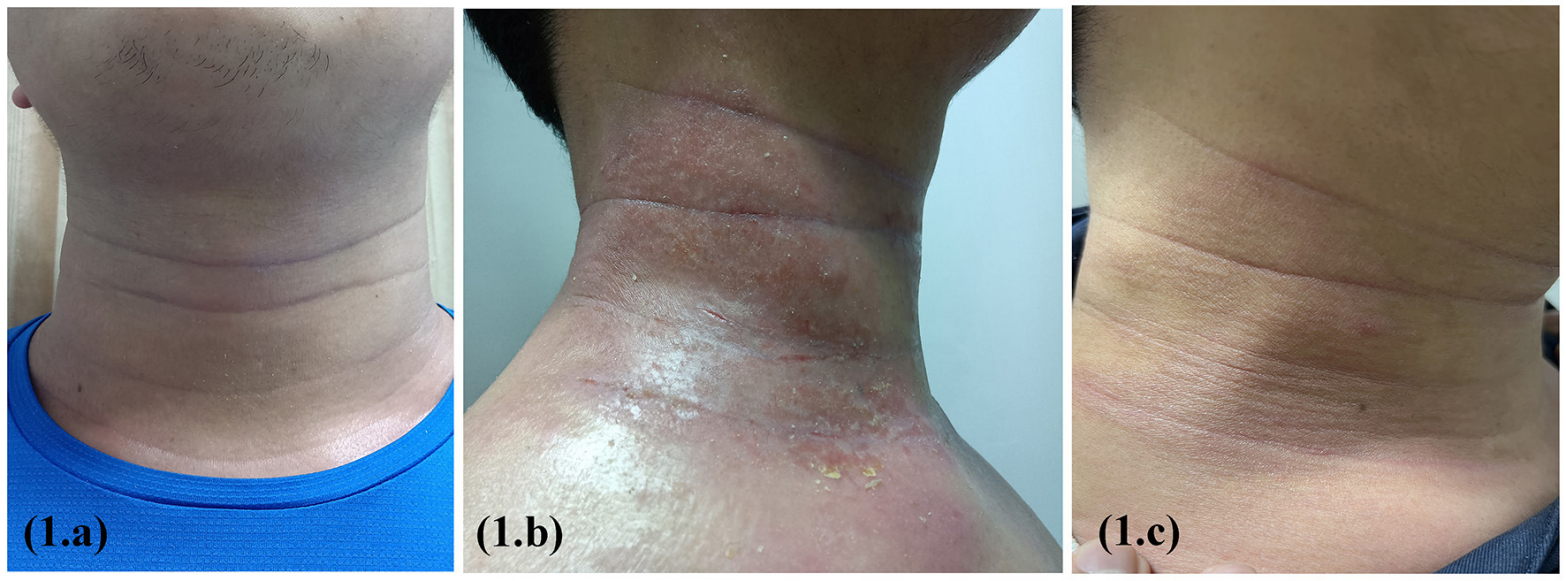
Erythema, scaling, and lichenification of the face and neck before dupilumab treatment **(a)**; obvious fissures, erosions, and purulent exudates in the patient's neck folds after 16 weeks on treatment **(b)**; the neck skin infection had significantly improved after 2 weeks of supportive therapy **(c)**.

A 17-year-old boy with a 6-year history of AD presented with xerosis, pruritus, and persistent eczema that had been resistant to traditional therapeutic approaches. Physical examination findings included a degree of erythema, scaling, and lichenification of the face and neck (Figure 1a), trunk, and upper and lower limbs. The patient otherwise was healthy with no other known systemic disease. The Score of Atopic Dermatitis (SCORAD) was 55, the Eczema Area and Severity Index (EASI) was 26, and the Pruritus Numerical Rating Scale (Pruritus-NRS) was 7/10. IgE > 2,000 kIU/l, blood eosinophil count: 8.6^*^109/l. On the basis of the medical history, clinical manifestations, laboratory test results, and diagnostic criteria of Hanifin and Rajka, the patient was diagnosed with AD. Dupilumab was administered at a loading dose of 600 mg followed by 300 mg biweekly. Photographs were obtained and assessed during the follow-up visits. Progressive improvement was observed in all lesions except the neck; however, progressive worsening of the paradoxical neck lesions was observed. At the 8th follow-up, there were obvious fissures, erosions, and purulent exudates in the patient's neck folds (Figure 1b). There was no history of other medications except dupilumab. The Score of Atopic Dermatitis (SCORAD) was 4, the Eczema Area and Severity Index (EASI) was 6, and the Pruritus Numerical Rating Scale (Pruritus-NRS) was 1/10. The fungal smear examination was negative, and local bacterial and fungal cultures and drug susceptibility tests showed *Staphylococcus aureus* infection and macrolide antibiotic sensitivity. The patient was administered local iodophor disinfection and oral azithromycin tablets. However, the dupilumab treatment was continued. At the 9th follow-up, the neck skin infection had significantly improved (Figure 1c).

### Case2

**Figure 2:**
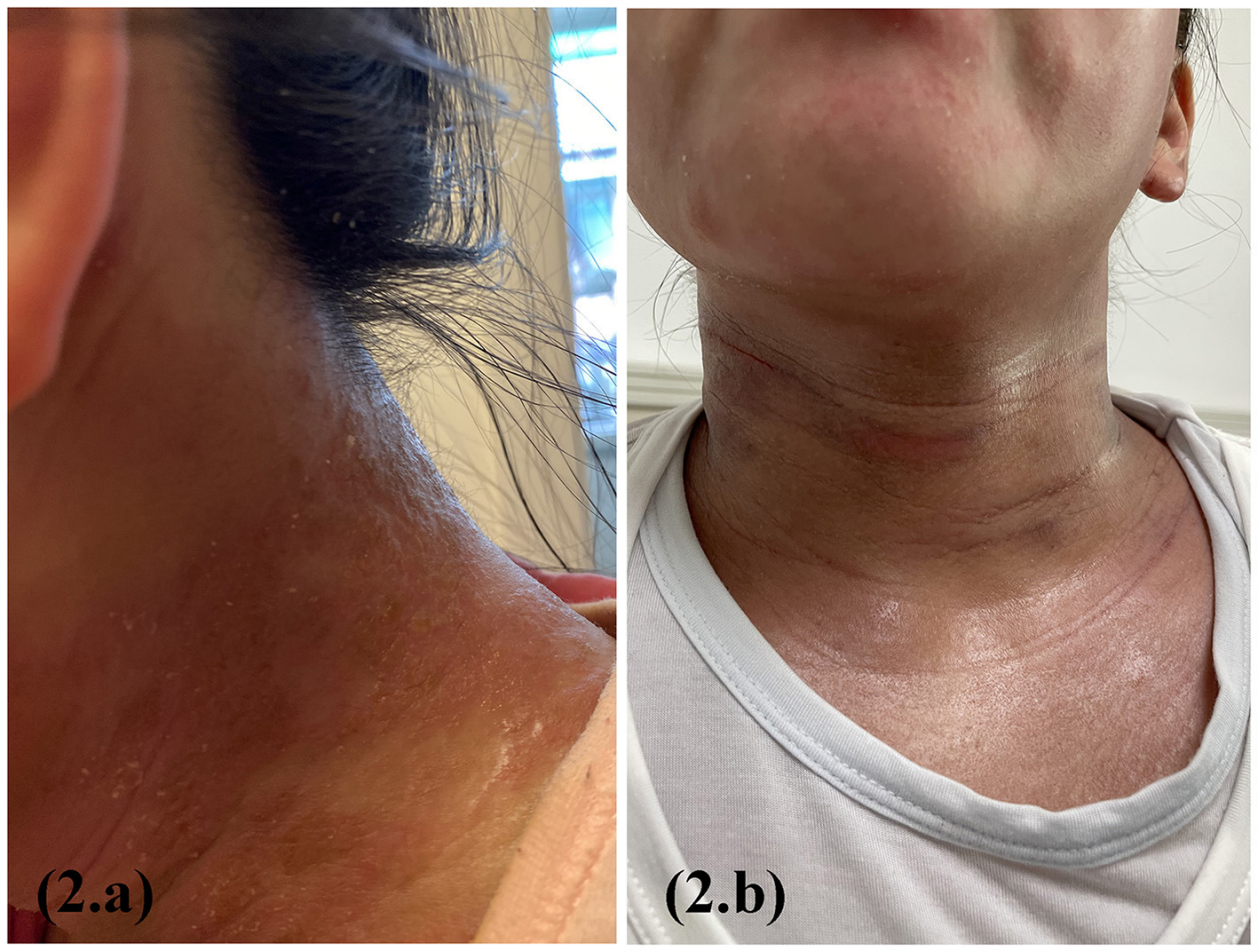
Erythema on the face and neck worsened on the second day after each dupilumab treatment **(a)**; 6 weeks after dupilumab discontinuation and supportive therapy **(b)**.

A 12-year-old girl with a 2-year history of AD presented with xerosis, erythematous papules on the flexors of the limbs and neck, and pruritus. The Score of Atopic Dermatitis (SCORAD) was 26, the Eczema Area and Severity Index (EASI) was 20, and the Pruritus Numerical Rating Scale (Pruritus-NRS) was 8/10. Traditional treatment was ineffective, and pruritus gradually worsened. Dupilumab was administered at a loading dose of 600 mg followed by 300 mg biweekly at another hospital. Based on the patient's recollection, erythema on the face and neck worsened on the second day after each dupilumab treatment; however, treatment was continued. After 6th dose, the symptoms got worse. Physical examination revealed generalized erythema with exudation and a tendency for erythroderma (Figure 2a). The Score of Atopic Dermatitis (SCORAD) was 90, the Eczema Area and Severity Index (EASI) was 60, and the Pruritus Numerical Rating Scale (Pruritus-NRS) was 10/10. Blood investigation results showed a white blood cell count of 10.0^*^109/l, eosinophil count of 3.1^*^109/l, and IgE 903 kIU/l. Bacterial and fungal cultures and drug susceptibility tests of the cervical exudate showed *Staphylococcus aureus* infection and macrolide sensitivity. The results of the fungal smear examination were negative. A biopsy revealed dermatitis and eczema-like changes. Improvements were observed after the discontinuation of dupilumab (Figure 2b). The patient was treated with local iodophor disinfection and oral azithromycin tablets and is still being followed up.

## Discussion

Atopic dermatitis is associated with disturbed microbiota, and *Staphylococcus aureus* has been identified as the major colonizer and pathogen (5). Atopic dermatitis is a chronic inflammatory skin disease, and the colonization rate of bacterial cultures on non-lesioned (39%) and damaged skin (70%) skin may affect up to 100% of these patients (6). The pathophysiology of atopic dermatitis is influenced by *Staphylococcus aureus* in a diverse range of ways, including barrier disruption and direct proinflammatory effects on type 2 immune activation (7).

A clinical trial showed that blocking IL-4Rα with dupilumab reduced *Staphylococcus aureus* colonization and increased microbial diversity in atopic dermatitis (4). Meta-analyses have suggested that the reduction in skin infections with dupilumab may be due to the normalization of the skin barrier and microbiome and the correction of abnormal immune responses in AD. Excessive IL-4 and IL-13 levels are associated with lower β-defensin levels; therefore, the dupilumab-induced reduction in IL-4 and IL-13 signaling may lead to increased β-defensin levels and a corresponding decrease in infection risk. Lower rates of skin infections are associated with an improvement in AD disease activity, rather than the specific effects of dupilumab (8).

Dupilumab is associated with a reduced risk of serious or severe infections and non-herpetic skin infections and does not increase overall infection rates compared to placebo in patients with moderate-to-severe AD (9). In this report, the skin lesions on the trunk and extremities significantly improved after dupilumab treatment, but the neck lesions aggravated, and *Staphylococcus aureus* infection occurred. Considering that paradoxical erythema only occurs in a typical neck distribution, we should consider Malassezia furfur-associated neck dermatitis, demodex-associated rosacea-like dermatosis, drug-induced photosensitivity reactions, alcohol-induced facial flushing, and allergic contact dermatitis (ACD). In our patients, fungal culture was negative, and no Demodex mites were observed under a microscope. Two patients did not consume alcohol or use photosensitizing drugs. In both clinical trials and real-world applications, the response to dupilumab treatment shows site heterogeneity, the response of skin lesions to the drug varies to some extent (10), and the efficacy of trunk and limb AD is significantly better than that of face and neck AD. Based on the above research results, *Staphylococcus aureus* infection can be considered an opportunistic infection caused by the colonization of *Staphylococcus aureus* in the poorly controlled site of AD. The aggravation of atopic dermatitis and *S. aureus* infection may be mutually causal, forming a vicious cycle.

Inhibition of the Th2 pathway by dupilumab hypothetically results in a shift toward a Th1-dominant response (11). An increasing number of patients report Th1-mediated skin disorders, such as exacerbated Th1-dependent allergic contact dermatitis (ACD), after the initiation of dupilumab (12). Dupilumab did not appear to have exert a dampening effect on the patch test results. AD with comorbid ACD is highly prevalent, and allergen avoidance results in a significant improvement in residual dermatitis that has not resolved without dupilumab therapy (13). This may be the reason for the increased incidence of neck erythema and concurrent *Staphylococcus aureus* infections. At present, there is no explanation for this phenomenon from the perspective of innate immunity, such as whether there is a drift in the inflammatory pathway related to *Staphylococcus aureus* infectivity. It has been postulated that during the chronic phase of AD, high IL-22 and low IL-17 expression predominates, and the production of antimicrobial peptides (AMP) is influenced by IL-22 (14). Dupilumab potentially shifts toward a more Th22-dominated response by inhibiting the Th2 pathway (15). This increases IL-22 levels, leading to a decrease in antimicrobial peptides and promotion of *S. aureus infection*.

It is worth considering that while dupilumab theoretically reduces *Staphylococcus aureus* colonization and increases microbial diversity in atopic dermatitis, it may vary by site in the real world. No studies have detected and compared the abundance of *S. aureus* in the different body parts of patients with AD. Dupilumab treatment results in a poor response to head, face, and neck rashes (10). Dupilumab is used, and strategies to reduce the burden of *S. aureus* on refractory areas such as the head, face, and neck need to be developed to minimize the pain and potential discontinuation of dupilumab treatment.

## Data availability statement

The original contributions presented in the study are included in the article/supplementary material, further inquiries can be directed to the corresponding author.

## Ethics statement

Ethical review and approval was not required for the study on human participants in accordance with the local legislation and institutional requirements. Written informed consent to participate in this study was provided by the participants' legal guardian/next of kin. Written informed consent was obtained from the participant/patient(s) for the publication of this case report.

## Author contributions

MC and KA wrote the original article, which KG edited and corrected. TX and YQ done data collection. JS and YY collected patients' photographs. LW provided article ideas and reviewed the article. All authors contributed to the article and approved the submitted version.
